# Publisher Correction: TGF-β blockade drives a transitional effector phenotype in T cells reversing SIV latency and decreasing SIV reservoirs in vivo

**DOI:** 10.1038/s41467-024-52897-z

**Published:** 2024-10-10

**Authors:** Jinhee Kim, Deepanwita Bose, Mariluz Araínga, Muhammad R. Haque, Christine M. Fennessey, Rachel A. Caddell, Yanique Thomas, Douglas E. Ferrell, Syed Ali, Emanuelle Grody, Yogesh Goyal, Claudia Cicala, James Arthos, Brandon F. Keele, Monica Vaccari, Ramon Lorenzo-Redondo, Thomas J. Hope, Francois Villinger, Elena Martinelli

**Affiliations:** 1 Department of Medicine, Division of Infectious Diseases, Feinberg School of Medicine, Northwestern University, Chicago, IL USA; 2 New Iberia Research Center, University of Louisiana at Lafayette, New Iberia, LA USA; 3 Cell and Developmental Biology, Feinberg School of Medicine, Northwestern University, Chicago, IL USA; 4 AIDS and Cancer Virus Program, Frederick National Laboratory for Cancer Research, Frederick, MD USA; 5 Division of Immunology, Tulane National Primate Research Center, Covington, LA USA; 6 Center for Synthetic Biology, Northwestern University, Chicago, IL USA; 7 Robert H. Lurie Comprehensive Cancer Center, Feinberg School of Medicine, Northwestern University, Chicago, IL USA; 8 Laboratory of Immunoregulation, National Institute of Allergy and Infectious Diseases, National Institutes of Health, Bethesda, MD USA; 9 Department of Microbiology and Immunology, Tulane University School of Medicine, New Orleans, LA USA; 10 Center for Pathogen Genomics and Microbial Evolution, Northwestern University Havey Institute for Global Health, Chicago, IL USA

**Keywords:** HIV infections, Immunotherapy, Translational research

Correction to: *Nature Communications* 10.1038/s41467-024-45555-x, published online 14 February 2024

The original version of the Peer Review File associated with this Article was updated after publication to redact the name of a researcher who prefers to remain anonymous. The file has been corrected.

