# Supplementary material for: TGF-β blockade drives a transitional effector phenotype in T cells reversing SIV latency and decreasing SIV reservoirs in vivo
Source: Nat Commun. 2024 Feb 14;15:1348. doi: 10.1038/s41467-024-45555-x (PMC10867093; doi:10.1038/s41467-024-45555-x)
Supplement: Supplementary file 3 — Description of Additional Supplementary Files [file 41467_2024_45555_MOESM3_ESM.pdf]

Supplementary Data 1

Hematology panel before and after infection, during ART and before and after each cycle of galunisertib.

Supplementary Data 2

Comprehensive metabolic panel (chemistry) before and after infection, during ART and before and after each cycle of galunisertib.

Supplementary Data 3

Raw data from flow cytometry classical analysis and MFIs

Supplemental Movie Legends.

Supplemental Movie 1. Timeline maximum intensity projections (MIP) of fused PET and CT scans are shown for 08M134 before and after each of the first 3 galunisertib cycle. Fusions of the whole-body CT and PET scans were generated for each timepoint. MIPs were generated using the MIM software and set to a numerical scale of 0-1.5 SUVbw visualized with the color scale PET Rainbow. The MIP were scaled to the same physical size using the dimensions to the scanner's visible range, and the scanner bed was removed from the CT scan. The MIP are displayed in chronological order, qualitatively demonstrating the changes in intensity and location of PET signals. Each movie generated in MIM as a series of MIP for one full rotation (~7 seconds) was captured using QuickTime.

Supplemental Movie 2. Timeline maximum intensity projections (MIP) of fused PET and CT scans are shown for 08M156 before and after each of the first 3 galunisertib cycle. Fusions of the whole-body CT and PET scans were generated for each timepoint. MIPs were generated using the MIM software and set to a numerical scale of 0-1.5 SUVbw visualized with the color scale PET Rainbow. The MIP were scaled to the same physical size using the dimensions to the scanner's visible range, and the scanner bed was removed from the CT scan. The MIP are

displayed in chronological order, qualitatively demonstrating the changes in intensity and location of PET signals. Each movie generated in MIM as a series of MIP for one full rotation (~7 seconds) was captured using QuickTime.

Supplemental Movie 3. Timeline maximum intensity projections (MIP) of fused PET and CT scans are shown for A6X003 before and after each of the first 3 galunisertib cycle. Fusions of the whole-body CT and PET scans were generated for each timepoint. MIPs were generated using the MIM software and set to a numerical scale of 0-1.5 SUVbw visualized with the color scale PET Rainbow. The MIP were scaled to the same physical size using the dimensions to the scanner's visible range, and the scanner bed was removed from the CT scan. The MIP are displayed in chronological order, qualitatively demonstrating the changes in intensity and location of PET signals. Each movie generated in MIM as a series of MIP for one full rotation (~7 seconds) was captured using QuickTime.

Supplemental Movie 4. Timeline maximum intensity projections (MIP) of fused PET and CT scans are shown for A8L014 before and after each of the first 3 galunisertib cycle. Fusions of the whole-body CT and PET scans were generated for each timepoint. MIPs were generated using the MIM software and set to a numerical scale of 0-1.5 SUVbw visualized with the color scale PET Rainbow. The MIP were scaled to the same physical size using the dimensions to the scanner's visible range, and the scanner bed was removed from the CT scan. The MIP are displayed in chronological order, qualitatively demonstrating the changes in intensity and location of PET signals. Each movie generated in MIM as a series of MIP for one full rotation (~7 seconds) was captured using QuickTime.

Supplemental Movie 5. Timeline maximum intensity projections (MIP) of fused PET and CT scans are shown for A8L057 before and after each of the first 3 galunisertib cycle. Fusions of the whole-body CT and PET scans were generated for each timepoint. MIPs were generated using the MIM software and set to a numerical scale of 0-1.5 SUVbw visualized with the color

scale PET Rainbow. The MIP were scaled to the same physical size using the dimensions to the scanner’s visible range, and the scanner bed was removed from the CT scan. The MIP are displayed in chronological order, qualitatively demonstrating the changes in intensity and location of PET signals. Each movie generated in MIM as a series of MIP for one full rotation (~7 seconds) was captured using QuickTime.

Supplemental Movie 6. Timeline maximum intensity projections (MIP) of fused PET and CT scans are shown for A8R095 before and after each of the first 3 galunisertib cycle. Fusions of the whole-body CT and PET scans were generated for each timepoint. MIPs were generated using the MIM software and set to a numerical scale of 0-1.5 SUVbw visualized with the color scale PET Rainbow. The MIP were scaled to the same physical size using the dimensions to the scanner’s visible range, and the scanner bed was removed from the CT scan. The MIP are displayed in chronological order, qualitatively demonstrating the changes in intensity and location of PET signals. Each movie generated in MIM as a series of MIP for one full rotation (~7 seconds) was captured using QuickTime.

Supplemental Movie 7. Timeline maximum intensity projections (MIP) of fused PET and CT scans are shown for 08T010 before and after each of the first 3 galunisertib cycle. Fusions of the whole-body CT and PET scans were generated for each timepoint. MIPs were generated using the MIM software and set to a numerical scale of 0-1.5 SUVbw visualized with the color scale PET Rainbow. The MIP were scaled to the same physical size using the dimensions to the scanner’s visible range, and the scanner bed was removed from the CT scan. The MIP are displayed in chronological order, qualitatively demonstrating the changes in intensity and location of PET signals. Each movie generated in MIM as a series of MIP for one full rotation (~7 seconds) was captured using QuickTime.

Supplemental Movie 8. Timeline maximum intensity projections (MIP) of fused PET and CT scans are shown for 08M171 before and after each of the first 3 galunisertib cycle. Fusions

of the whole-body CT and PET scans were generated for each timepoint. MIPs were generated using the MIM software and set to a numerical scale of 0-1.5 SUVbw visualized with the color scale PET Rainbow. The MIP were scaled to the same physical size using the dimensions to the scanner's visible range, and the scanner bed was removed from the CT scan. The MIP are displayed in chronological order, qualitatively demonstrating the changes in intensity and location of PET signals. Each movie generated in MIM as a series of MIP for one full rotation (~7 seconds) was captured using QuickTime.

Supplemental Movie 9. Representative regions drawn to generate the gut ROI for A8L014. Representative example of MIP serie generated in MIM for full rotation including ROIs as different colored lines for 1 monkey at 1 time point. The gut region (yellow line) was identified from beneath the stomach down to the cervix on the coronal view. To isolate the gut ROI, specific exclusions were made using Boolean gates to remove the kidneys (purple line), liver (blue line), spleen (red line), and bones (green line) along with their associated signals from within the gut area.

Supplemental Movie 10. Representative regions drawn to generate the LN ROI. Representative example of MIP series of Axial view depicting axillary lymph nodes ROI for all scans of a single macaque (A8R057). The Axillary level 1 was contoured on the axial plane of the time point with the most robust signal on each side and transferred to other time points using a multi-time-point workflow in MIM. The data from each side were averaged (SUVmean) or combined (SUVtotal).
