# Supplementary material for: TGF-β blockade drives a transitional effector phenotype in T cells reversing SIV latency and decreasing SIV reservoirs in vivo
Source: Nat Commun. 2024 Feb 14;15:1348. doi: 10.1038/s41467-024-45555-x (PMC10867093; doi:10.1038/s41467-024-45555-x)
Supplement: Supplementary file 17 — Reporting Summary [file 41467_2024_45555_MOESM17_ESM.pdf]

nature portfolio

Corresponding author(s): Elena Martinelli

Last updated by author(s): Jan 11 2024

Reporting Summary

Nature Portfolio wishes to improve the reproducibility of the work that we publish. This form provides structure for consistency and transparency in reporting. For further information on Nature Portfolio policies, see our Editorial Policies and the Editorial Policy Checklist.

Please do not complete any field with "not applicable" or n/a. Refer to the help text for what text to use if an item is not relevant to your study.
For final submission: please carefully check your responses for accuracy; you will not be able to make changes later.

Statistics

For all statistical analyses, confirm that the following items are present in the figure legend, table legend, main text, or Methods section.

n/a	Confirmed	
☐	☒	The exact sample size (*n*) for each experimental group/condition, given as a discrete number and unit of measurement
☐	☒	A statement on whether measurements were taken from distinct samples or whether the same sample was measured repeatedly
☐	☒	The statistical test(s) used AND whether they are one- or two-sided<br>*Only common tests should be described solely by name; describe more complex techniques in the Methods section.*
☐	☒	A description of all covariates tested
☐	☒	A description of any assumptions or corrections, such as tests of normality and adjustment for multiple comparisons
☐	☒	A full description of the statistical parameters including central tendency (e.g. means) or other basic estimates (e.g. regression coefficient) AND variation (e.g. standard deviation) or associated estimates of uncertainty (e.g. confidence intervals)
☐	☒	For null hypothesis testing, the test statistic (e.g. *F*, *t*, *r*) with confidence intervals, effect sizes, degrees of freedom and *P* value noted<br>*Give P values as exact values whenever suitable.*
☒	☐	For Bayesian analysis, information on the choice of priors and Markov chain Monte Carlo settings
☒	☐	For hierarchical and complex designs, identification of the appropriate level for tests and full reporting of outcomes
☐	☒	Estimates of effect sizes (e.g. Cohen's *d*, Pearson's *r*), indicating how they were calculated

Our web collection on statistics for biologists contains articles on many of the points above.

Software and code

Policy information about availability of computer code

Data collection	Diva v6 software was used for data collection of flow cytometry data.
Data analysis	Flowjo v10 was used for flow cytometry analysis. For the high-dimensional data analysis, tSNE and flowSOM plugins in Flowjo v10 were utilized, and figures and statistics were generated in Python. Partek Flow genomic analysis suite, in combination with Parse Bioscience proprietary scripts, was used for scRNAseq analysis. For bulk RNA-seq analysis, Trimmomatic-0.36 and Hisat2-Stringtie pipeline were employed for primary analysis. Differential expression analysis was conducted using DESeq2 and gene set enrichment analysis was performed using GSEA and Metascape. MIM software was used for PET/CT images analysis.

For manuscripts utilizing custom algorithms or software that are central to the research but not yet described in published literature, software must be made available to editors and reviewers. We strongly encourage code deposition in a community repository (e.g. GitHub). See the Nature Portfolio guidelines for submitting code & software for further information.

Data

Policy information about availability of data

All manuscripts must include a data availability statement. This statement should provide the following information, where applicable:

- Accession codes, unique identifiers, or web links for publicly available datasets
- A description of any restrictions on data availability
- For clinical datasets or third party data, please ensure that the statement adheres to our policy

All relevant data are included in the manuscript or supplemental material. Raw data files including DICOM image files are available to be shared upon request to the corresponding author elena.martinelli@northwestern.edu. All RNA sequencing data originating from this study have been deposited in NCBI GEO under the accession code: GSE244871

Research involving human participants, their data, or biological material

Policy information about studies with human participants or human data. See also policy information about sex, gender (identity/presentation), and sexual orientation and race, ethnicity and racism.

Reporting on sex and gender	N/A
Reporting on race, ethnicity, or other socially relevant groupings	N/A
Population characteristics	N/A
Recruitment	N/A
Ethics oversight	N/A

Note that full information on the approval of the study protocol must also be provided in the manuscript.

Field-specific reporting

Please select the one below that is the best fit for your research. If you are not sure, read the appropriate sections before making your selection.

☒ Life sciences ☐ Behavioural & social sciences ☐ Ecological, evolutionary & environmental sciences

For a reference copy of the document with all sections, see nature.com/documents/nr-reporting-summary-flat.pdf

Life sciences study design

All studies must disclose on these points even when the disclosure is negative.

Sample size	Macaques for research are an extremely valuable resource. Hence, every effort was made to minimize the number of animals used in this study. n=8 for the study group was chosen because considered to have an acceptable 80% power to detect an effect size of dz=1.19
Data exclusions	All data were included in the analysis, but for imaging data of 08M171 in Figure 2 as indicated in the article
Replication	In addition to the biological replicates every assay was run with 2 or 3 technical replicates. All attempt at replication were successful
Randomization	The study has only 1 group of animals treated with the drug. Analsyis is before vs after
Blinding	The assay performer was blind to the time point (eg before or after drug)

Behavioural & social sciences study design

All studies must disclose on these points even when the disclosure is negative.

Study description	N/A
Research sample	N/A
Sampling strategy	N/A
Data collection	N/A
Timing	N/A
Data exclusions	N/A
Non-participation	N/A
Randomization	N/A

Ecological, evolutionary & environmental sciences study design

All studies must disclose on these points even when the disclosure is negative.

Study description

Research sample

Sampling strategy

Data collection

Timing and spatial scale

Data exclusions

Reproducibility

Randomization

Blinding

Did the study involve field work? ☐ Yes ☐ No

Field work, collection and transport

Field conditions

Location

Access & import/export

Disturbance

Reporting for specific materials, systems and methods

We require information from authors about some types of materials, experimental systems and methods used in many studies. Here, indicate whether each material, system or method listed is relevant to your study. If you are not sure if a list item applies to your research, read the appropriate section before selecting a response.

Materials & experimental systems

n/a	Involved in the study	
☐	☒	Antibodies
☒	☐	Eukaryotic cell lines
☒	☐	Palaeontology and archaeology
☐	☒	Animals and other organisms
☒	☐	Clinical data
☒	☐	Dual use research of concern
☒	☐	Plants

Methods

n/a	Involved in the study	
☒	☐	ChIP-seq
☐	☒	Flow cytometry
☒	☐	MRI-based neuroimaging

Antibodies

Antibodies used

PBMC High-Dim
DAPI FVS440UV Live Dead BD
BUV395 CD4 L200 BD
BUV496
BUV563 CD8 RPA-T8 BD
BUV615
BUV661 CCR7 3D12 BD
BUV737 CD16 3G8 BD
BUV805 CD3 SP34-2 BD
BV421 CD73
BV480 Ki67** B56 BD
BV510 CD28 28.2 Biolegend
BV570
BV605 CD69 FN50 Biolegend
BV650 HLA-DR L243 BD
BV711 CD62L SK11 BD
BV750 CD45RA 5H9 BD
BV786 Tbet 4B10 Biolegend
AF488 CCR6 G034E3 Biolegend
BB630 Tim-3 7D3 BD
BB660/PCP
BB700/PCPeFluo710 PD-1 eBioJ105 eBioscience
BB755
BB790 GRZB** GB11 BD
PE TCF-1** S33-966 BD
PE-CF594 CD95 DX2 Biolegend
PE-Vio770 NKG2A REA110 Mylteny
APC a4b7 Act-1 NHP
AF700/R718 CD101 V7.1 BD
APC-Fire750 CD56 HCD56 Biolegend

Treg/Tfh Panel
FITC FOXP3 PCH101
EBioscience
PCP-Cy5.5 CD4 L200 BD
BV421 CTLA4 BNI3
Biolegend
BV510 L/D N/A BD
BV605 CD95 DX2 Biolegend
BV650 PD-1 EH12.2H7 Biolegend
BV711 CD39 A1 Biolegend
BUV395 CD25 BC96 BD
BUV737 CD28 CD28.2 BD
BUV805 CD8 RPA-T8 Biolegend
PE-Cy5 CXCR5 C398.4A Biolegend
PE-Cy7 CCR4 L291H4 Biolegend
APC TCF-1 s33-966(RUO) BD
AL700 CD3 SP34-2 BD

Validation

All antibodies were tested and titrated in Martinelli's laboratory for cross reactivity with macaque cells.
All antibodies were from commercial sources (indicated in Table S4) providing on their website extensive validation data (in human and/or macaque cells)

Eukaryotic cell lines

Policy information about cell lines and Sex and Gender in Research

Cell line source(s)	No cell lines were used
Authentication	
Mycoplasma contamination	
Commonly misidentified lines (See ICLAC register)	

Palaeontology and Archaeology

Specimen provenance	
Specimen deposition	
Dating methods	

☐ Tick this box to confirm that the raw and calibrated dates are available in the paper or in Supplementary Information.

Ethics oversight	

Note that full information on the approval of the study protocol must also be provided in the manuscript.

Animals and other research organisms

Policy information about studies involving animals; ARRIVE guidelines recommended for reporting animal research, and Sex and Gender in Research

Laboratory animals	Indian origin rhesus macaques (Macaca Mulatta, Mamu-A01-, -B08, -B17-, all females) Age is indicated in Table S1
Wild animals	No wild animals were used in the study
Reporting on sex	Only female macaques were used in this study because of restricted availability.
Field-collected samples	No field-collected samples were used in the study
Ethics oversight	The study was approved by the Institutional Animal Care and Usage Committees (IACUC) of the University of Louisiana at Lafayette (2021-8821-002; protocol 8821-01).

Note that full information on the approval of the study protocol must also be provided in the manuscript.

Clinical data

Policy information about clinical studies

All manuscripts should comply with the ICMJE guidelines for publication of clinical research and a completed CONSORT checklist must be included with all submissions.

Clinical trial registration	
Study protocol	
Data collection	
Outcomes	

Dual use research of concern

Policy information about dual use research of concern

Hazards

Could the accidental, deliberate or reckless misuse of agents or technologies generated in the work, or the application of information presented in the manuscript, pose a threat to:

No | Yes

- [x] No [ ] Yes — Public health
- [x] No [ ] Yes — National security
- [x] No [ ] Yes — Crops and/or livestock
- [x] No [ ] Yes — Ecosystems
- [x] No [ ] Yes — Any other significant area

Experiments of concern

Does the work involve any of these experiments of concern:

No | Yes

- [x] No [ ] Yes — Demonstrate how to render a vaccine ineffective
- [x] No [ ] Yes — Confer resistance to therapeutically useful antibiotics or antiviral agents
- [x] No [ ] Yes — Enhance the virulence of a pathogen or render a nonpathogen virulent
- [x] No [ ] Yes — Increase transmissibility of a pathogen
- [x] No [ ] Yes — Alter the host range of a pathogen
- [x] No [ ] Yes — Enable evasion of diagnostic/detection modalities
- [x] No [ ] Yes — Enable the weaponization of a biological agent or toxin
- [x] No [ ] Yes — Any other potentially harmful combination of experiments and agents

Plants

Seed stocks

Novel plant genotypes

Authentication

ChIP-seq

Data deposition

- [ ] Confirm that both raw and final processed data have been deposited in a public database such as GEO.
- [ ] Confirm that you have deposited or provided access to graph files (e.g. BED files) for the called peaks.

Data access links
May remain private before publication.

Files in database submission

Genome browser session
(e.g. UCSC)

Methodology

Replicates

Sequencing depth

Antibodies

Peak calling parameters

Data quality

Software

Flow Cytometry

Plots

Confirm that:

☒ The axis labels state the marker and fluorochrome used (e.g. CD4-FITC).

☒ The axis scales are clearly visible. Include numbers along axes only for bottom left plot of group (a 'group' is an analysis of identical markers).

☒ All plots are contour plots with outliers or pseudocolor plots.

☒ A numerical value for number of cells or percentage (with statistics) is provided.

Methodology

Sample preparation: PBMC were thawed before staining, permeabilization and fixation as indicated in the methods.

Instrument: FACS Symphony A5

Software: DIVA v6

Cell population abundance: No sorting occurred

Gating strategy: All cells were gated within live and singlet before proceeding with analysis. Gating strategy is included in the supplementary material

☒ Tick this box to confirm that a figure exemplifying the gating strategy is provided in the Supplementary Information.

Magnetic resonance imaging

Experimental design

Design type

Design specifications

Behavioral performance measures

Imaging type(s)

Field strength

Sequence & imaging parameters

Area of acquisition

Diffusion MRI ☐ Used ☐ Not used

Preprocessing

Preprocessing software

Normalization

Normalization template

Noise and artifact removal

Volume censoring

Statistical modeling & inference

Model type and settings

Effect(s) tested

Specify type of analysis: ☐ Whole brain ☐ ROI-based ☐ Both

Statistic type for inference

(See Eklund et al. 2016)

Correction

Models & analysis

n/a	Involved in the study
☐	☐ Functional and/or effective connectivity
☐	☐ Graph analysis
☐	☐ Multivariate modeling or predictive analysis

Functional and/or effective connectivity

Graph analysis

Multivariate modeling and predictive analysis

This checklist template is licensed under a Creative Commons Attribution 4.0 International License, which permits use, sharing, adaptation, distribution and reproduction in any medium or format, as long as you give appropriate credit to the original author(s) and the source, provide a link to the Creative Commons license, and indicate if changes were made. The images or other third party material in this article are included in the article's Creative Commons license, unless indicated otherwise in a credit line to the material. If material is not included in the article's Creative Commons license and your intended use is not permitted by statutory regulation or exceeds the permitted use, you will need to obtain permission directly from the copyright holder. To view a copy of this license, visit http://creativecommons.org/licenses/by/4.0/
